# Water Is an Active Element: A Randomized Double-Blind Controlled Clinical Trial Comparing Cutaneous Lipidomics in Consumers Drinking Two Different Bicarbonate-Calcic Waters (Medium-Mineral vs. Oligo-Mineral)

**DOI:** 10.3390/biomedicines11041036

**Published:** 2023-03-27

**Authors:** Giovanni Damiani, Ilaria Controne, Hilmi Al-Shakhshir, Paolo D. M. Pigatto

**Affiliations:** 1 Clinical Dermatology, IRCCS Istituto Ortopedico Galeazzi, 20161 Milan, Italy; 2 Department of Biomedical, Surgical and Dental Sciences, University of Milan, 20122 Milan, Italy; 3 Department of Dermatology, Case Western Reserve University, Cleveland, OH 44106, USA; 4 Italian Center of Precision Medicine and Chronic Inflammation, 20122 Milan, Italy

**Keywords:** oligo-mineral water, medium-mineral water, waters, skin, precision medicine, lipidomics

## Abstract

Despite the well-known cutaneous beneficial effect of thermal water on the skin, no data exist regarding the potential biological effect of orally consumed water on healthy skin. Thus, in this single-center, double-blind, randomized controlled clinical trial conducted on age and menstrual cycle timing-matched healthy female volunteers (24 + 24) consuming water A (oligo-mineral) or water B (medium-mineral) for 1 month (T1), the cutaneous lipidomics were compared. Interestingly, only water A consumers had a statistically significant (*p* < 0.001) change in cutaneous lipidomics, with 66 lipids different (8 decreased and 58 increased). The cutaneous lipidomics of consumers of water A vs. water B were statistically different (*p* < 0.05). Twenty cutaneous lipids were necessary to predict the water type previously consumed (AUC ~70). Our study suggests that drinking oligo-mineral water may change skin biology and may influence the cutaneous barrier, so future dermatological clinical trials should also account for the water type consumed to avoid potential confounders.

## 1. Introduction

Ablutions in thermal water are an ancient treatment for several dermatological disorders refractory to the poor topical pharmacopeia that shamans, healers, alchemists, surgeons and doctors worldwide adopted [1,2,3,4,5].

Preliminary in vitro studies on keratinocyte cultures suggested antioxidant [6], anti-angiogenic [7] and anti-inflammatory [6] properties clinically interpreted as a global anti-senescence effect in healthy skin. Furthermore, these characteristics are also beneficial as a complementary contact treatment for pathological cutaneous conditions, ranging from inflammatory (i.e., psoriasis) [4] to allergic (i.e., atopic dermatitis) [8]. Using modelling, Caiazzo et al. recently evaluated the effect of daily ultraviolet cutaneous exposure in HaCaT keratinocytes, finding that oligo-mineral water was protective and limited UVB-related pro-inflammatory effects [9].

Water can also be classified using the fixed residue (fd) of mineralized, oligo-mineral, mineral or medium-mineral, and highly mineralized (see Section 2.1.1. for more details). Fd contributes to the taste of water and, consequently, also to consumer preferences; for example, in Italy, >90% of the water consumed daily belongs to the oligo-mineral and medium-mineral categories.

Interestingly, evidence for the potential cutaneous effects of water consumed orally is widespread and seldom discordant [10,11,12], especially in healthy subjects. Moreover, encouraging results are displayed in patients with atopic dermatitis [13].

Although no linear correlation has been proved between water intake and skin hydration, some studies sustained that proper water intake may also marginally improve skin hydration in healthy patients [14,15]. Remarkably, water is still regarded as an inert drink, not meritorious to be standardized in nutritional, as well as clinical, studies, in plenty contradiction to its effect on vessels [12,16].

Thus, we decided to design a clinical trial to evaluate the effect of different water types on skin biology in healthy patients.

## 2. Materials and Methods

### 2.1. Study Design

This is a single-center, double-blind (subject and investigator), randomized controlled clinical trial conducted on healthy volunteers. The study includes age and menstrual cycle timing-matched female subjects who blindly consumed water A or water B, both marketed as food and not supplements. The enrolled subjects were asked to drink 2 L of water of the assigned type (water A or water B) per day.

At the baseline (T0) and after 1 month (T1), patients (a) were clinically evaluated independently by two board-certified dermatologists (>5 years of experience), (b) performed complete blood count (CBC), creatinine and physical (macroscopic) chemical urine tests, and (c) collected a skin stripping with Patch Sebutape^®^.

The study fulfilled the Helsinki Declaration and the General Data Protection Regulation 2016/679, so it was approved by the Institutional Review Board of San Raphael Hospital (protocol code: 176/INT/2020 and date of approval: 11 November 2020). All participants signed an approved informed consent form.

#### 2.1.1. Mineral Water Evaluation

Waters were classified in accord with the Marotta & Sicca classification of Italian mineral waters that remains the parameters required by Italian law to market mineral waters (see D.Lgs. 25.01.1992 n°105 and D.Lgs. 176/2011) [17]. The parameters evaluated in the algorithm are temperature, fixed residue (fd) and chemical composition. With respect to the temperature, mineral waters can be divided into (a) cold, with a temperature < 20 °C; (b) hypothermal, with a temperature between 20 °C and 30 °C; (c) homeothermal or simply thermal, with a temperature between 30 °C and 40 °C; and (d) hyperthermal, with a temperature > 40 °C. With respect to fd, mineral waters are differentiated by the categories: (a) mineralized, with fd < 50 mg/L; (b) oligo-mineral, with fd < 500 mg/L; (c) mineral or medium-mineral, with fd between 500 and 1000 mg/L; and (d) highly mineralized, with fd > 1500 mg/L. Then, based on the chemical composition of the fixed residue, thermal water derives its name from the element or set of elements from which it is formed. Parameters important in this phase are the main anion (or anions) and main cation; when an ion is present in quantities greater than 20 mEq/L, it gives the name to the water. Based on the prevailing ionic composition, mineral waters are classified into (a) bicarbonate, (b) sauce or chloride-sodic, (c) sulfuric, (d) ferruginous, (e) arsenicated and (f) sulphated.

Notably, chemical analyses of water A (bicarbonate-calcic oligo-mineral, marketed as Rocchetta water) and B (bicarbonate-calcic medium-mineral) are reported in Supplementary S1.

### 2.2. Inclusion and Exclusion Criteria

We enrolled (a) female subjects, (b) aged between 30 and 50 years, (c) in good health (absence of pathologies that have an International Classification of Diseases (ICD) code in compliance with the definition of the World Health Organization: “a state of complete physical, mental and social well-being and not merely the absence of disease or infirmity.”), (d) omnivores [18,19,20,21,22,23], (e) Italian native speakers, (f) who signed the informed consent form.

Conversely, subjects were excluded if they were/had (a) males; (b) in a different age range (<30 and >50 years of age) or with a Body Mass Index (BMI) < 19 or >30; (c) active or latent inflammatory, oncological and rheumatological diseases; (d) menopausal/gynecological disorders (i.e., dysmenorrhea); (e) chronic dermatoses (i.e., psoriasis or even a transitory dermatosis in the previous 6 months); (f) allergic disorders (i.e., asthma [24], atopic dermatitis [25,26] or multiple chemical sensitivity (MCS) [27]); (g) a different diet than omnivore, included fasting; (h) active renal/urological disorders, or even a history thereof; (i) refused to sign the informed consent form.

### 2.3. Dermatological Evaluation

Medical histories were carefully collected by two Italian, experienced (>5 years of experience in university hospitals), board-certified dermatologists (G.D. and P.P.). Clinical and dermatoscopic evaluations were performed at T0 and T1.

Dermatologists also administered the Skin Satisfaction Questionnaire (SSQ) at T0 and T1 to the enrolled subjects [28].

### 2.4. Sample Processing

The cutaneous sample was obtained with Patch Sebutape^®^ (Cantabria Labs, Varese, Italy) placed on the cleansed (micellar water + clorexidin 2%) glabella region for 30 min, and after removal, it was stocked in a 13 mL vial. Then, we added 5 mL dichloromethane:methanol (2:1), and the vial was shook for 15 s with a vortex. We performed a sonication for 5 min and a centrifugation at 4000 rpm for 10 min at 15 °C on the solution of the organic phase, which was transferred to an Eppendorf tube and concentrated under nitrogen flow until the extract was completely dry. The residue was centrifuged for 5 min at 4000 rpm at 15 °C, and the supernatant was collected and finally analyzed. At the same time as the analysis of the samples, QC samples obtained by taking and combining 15 µL of each extracted sample were prepared and analyzed.

### 2.5. High-Resolution Mass Spectrometry Analysis (UHPLC-HRMS)

All samples were analyzed at UNITECH OMICs (University of Milano, Italy) using an ExionLC™ AD system (SCIEX, AB Sciex Pte. Ltd., Singapore) connected to a TripleTOF™ 6600 System (SCIEX, AB Sciex Pte. Ltd., Singapore) equipped with a Turbo V™ Ion Source with an ESI Probe. The software used was SCIEX OS version 3.1 (AB Sciex Pte. Ltd., Singapore).

#### Chromatography of the Skin Strips

The chromatographic separation on a Kinetex^®^ EVO C18—2.1 × 100 mm, 1.7 μm (Phenomenex, Torrance, CA, USA) equipped with a precolumn was achieved using, as mobile phase A, water/acetonitrile (60:40) and, as mobile phase B, 2-propanol/acetonitrile (90:10), both containing 10 mM ammonium acetate and 0.1% of formic acid. The flow rate was 0.4 mL/min, and the column temperature was 45 °C. The elution gradient was set as 0–2 min (45% B), 2–12 min (45–97% B), 12–17 min (97% B), 17–17.10 min (97–45% B) and 17.10–20 min (45% B). The sample injection volume was 5 μL. Details are carefully reported in Table 1.

MS spectra of the skin was collected over an *m*/*z* range of 140–2000 Da in positive and negative polarity, operating in IDA mode (Information Dependent Acquisition, SCIEX). The collision energy was set at 35 (CES 15).

### 2.6. Data Analysis

The False Discovery Rate (FDR) was calculated with Tibshirani and Storey’s q-value, with a = 0.05 or b = 0.10. The True Discovery Rate (TDR) was estimated from 1000 series of simulations in R. The CV (coefficient of variation) for the simulation was set at 0.35. The importance of the single metabolite depends on the ratio between the log ((FC))/CV, in which FC increases or decreases in the single metabolite. We chose an FDR of 0.83, hypothesizing that 200 metabolites of concentrations of at least 1.5 times would truly differ. Thus, our sample size calculation suggested a minimum of 20 subjects for the cases and controls that we increased 20%, arriving at an enrollment of 48 (24 + 24) subjects.

Clinical variables’ distribution was assessed for normality by applying the Kolmogorov–Smirnov test. Data were computed as means ± standard deviations for continuous variables, and they were expressed as percentages in the case of categorical parameters. Student’s *t*-test for paired samples was applied to compute the mean differences before and after drinking Water type A or B.

All samples were processed with MS-DIAL software ver. 4.24 by setting the LipidBlast integrated database (version 68) (Fiehn Lab, UC Davis, Davis, CA, USA). The processing of the files was set by applying the normalization that supports the LOWESS algorithm and the “Blank Filter” option that filters the peaks present in the “Blank Sebutape” samples. Following this normalization, the program returned a number of identifications (IDs) based on the value of the *m*/*z* (parent ion) found and determined in high resolution of these IDs, those who, in the QC samples alone, had a CV% (calculated on the area value of each single ID) lower than 40%, with a number of identifications equal to thirty (*n* = 30). After this first processing of the samples under analysis, only the certain identifications (IDs) were considered by comparison with the LipidBlast database and with the acquired MS/MS spectrum.

Data for skin were obtained separately and analyzed using R version 4.0.3 (Lucent Technologies, Murray Hill, NJ, USA). Each dataset was cleaned and underwent quality control to ensure there were no outliers. Samples that had an “nd”, signifying not detected, were changed to 0.

Principal component analysis was performed on the datasets using the function prcomp(), with the options “center = TRUE” and “scale = TRUE”, from R package stats version 4.0.3. Plots were generated using R package ggplot2 version 3.3.6, while heatmaps were generated using pheatmap version 1.0.12.

Differential abundance analysis was performed using the wilcox.test() function from R package stats version 4.0.3. Given that we paired samples, Time 0 and Time 1 for each sample pair, in comparisons with paired samples, we used the option “paired = TRUE”; otherwise, comparisons between different groups used “paired = FALSE”. The correction for multiple testing was performed using the *p*.adjust() function using the Benjamini–Hochberg method. Samples with a PAdj ≤ 0.05 were considered significant; meanwhile, when there was a lack of any significant lipids, a *p* value ≤ 0.05 was used instead.

Using the cv.glmnet() function with “family = ‘binomial’” from R package glmnet version 4.1-2, a classifier using k-fold cross-validation was built to attempt to differentiate between the different types of water used. The area under the curve was calculated and used as the metric to determine the model that has the highest accuracy.

## 3. Results

### 3.1. Clinical Data

In the present clinical trial, 48 (24 + 24) female subjects were enrolled, and their mean age was 44.7 ± 2.6 years of age. No age differences were detected between Water A and Water B drinkers. The average BMI of the enrolled patients was 24.3 ± 2.6 kg/m^2^ (64.6 ± 8.1 kg), with no difference between the two considered groups (*p* < 0.05).

SSQ globally improved in both groups, but in a non-statistically significant manner (*p* > 0.05); interestingly, subjects that consumed Water A manifested a statistically significant decrease of SSQ from T0 to T1.

### 3.2. Differences in Cutaneous Lipidomics between the Two Groups Drinking Different Waters

Interestingly, after 1 month, only patients that consumed Water A displayed a change in cutaneous lipidomics. In total, 66 lipids registered a statistically significant change (*p* < 0.001): 54 Glycerolipids (18 Diacylglycerols (DG), 12 EtherDG, 24 Triacylglycerols (TG)), 8 Glycerophospholipids (5 Lysophophatidylcholines (LPC), 3 Phosphatidylcholines (PC)), 3 Sphingolipids, 1 Hexosylceramide hydroxyfatty acid-sphingosines (HexCer_HS), 1 Sphingosine (Sph), 1 Sphinganine (DHSph) and 1 Fatty acyls (1 Acylcarnitine (CAR)). Overall, 8 glycerolipids decreased (6 TG and 2 DG), and the others increased. For further details, see Table 2.

Subjects who consumed Water B did not display any statistically significant change in cutaneous lipidomics.

Comparing the T1 lipidomics in the two groups, the difference was nearly statistically significant, so we further evaluated the data with Principal Component Analysis.

**Figure 1:**
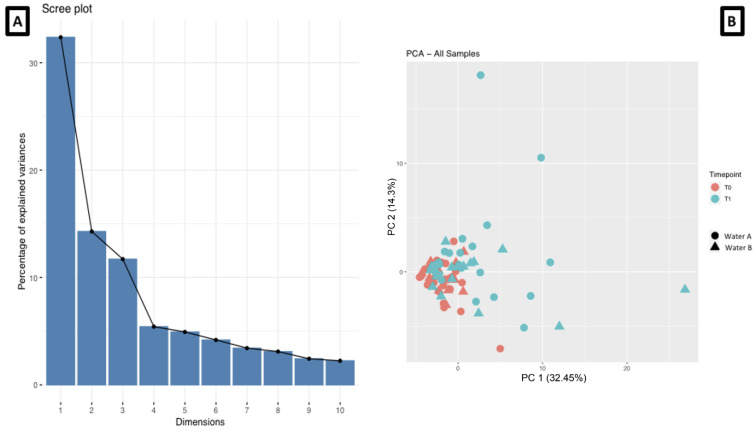
Cutaneous lipidomics evaluation of total variance (**A**) and representation of the two main Principal Component Analyses (**B**).

The first two PCAs were more representative of the data variance (Figure 1A), so we adopted and graphically represented it in Figure 1B (PC1 = 32.45% and PC2 = 14.3%). It suggested that a difference in the two groups was present, but linear statistics did not magnify it.

**Figure 2:**
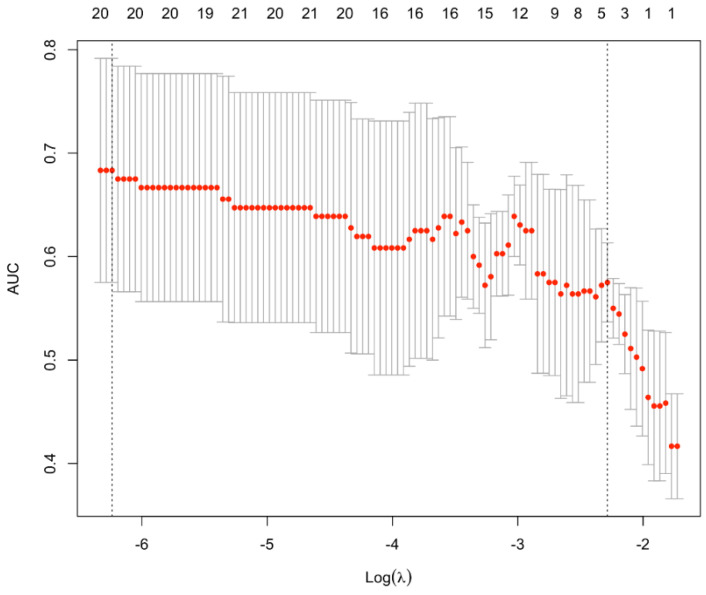
Accuracy of the predictive algorithm that uses cutaneous lipids to predict the water type consumed. The line composed by red dots depicts the average accuracy of the presented predictive algorithm. AUC: Area under the ROC Curve.

Thus, we decided to apply machine learning to predict which water was consumed, starting from the cutaneous lipids abundance at T1. Surprisingly, only 20 lipids were necessary to predict the water consumed, with an accuracy of almost 70% (Figure 2).

For the list of predictive lipids, see Table 3.

## 4. Discussion

In this clinical trial, we demonstrated that water orally consumed had an effect on skin lipidomics; oligo-mineral water has a prominent effect on cutaneous biology if compared with the medium-mineral one.

Some evidence evaluated the biological effect of water consumed orally on dermatologically healthy patients, but it focused mainly on cerebral circulation [16] or on pathological skin [13]. These aspects, together with the biologically relevant effect of oligo-mineral water, suggest that researchers should carefully mention the water type in the inclusion/exclusion criteria in observation studies and clinical trials examining nutrition in dermatology and urology.

Ideally, studies should include the water type orally consumed in the exposome to modify the cutaneous outcomes, especially in complex skin disorders, in which susceptible genes [29,30] interact with exposure to manifest the pathological phenotype (i.e., psoriasis or hidradenitis suppurativa) [31,32]. In fact, exposures may condition the epigenetics that modulate genes expression [33] and even drug response [34].

From this perspective, the water ingested should be regarded as a supplement, or even a drug, that synergically collaborates with drugs to improve the clinical outcome. The present evidence may also sustain, with solid evidence, that the water type orally consumed should be reported in the medical history, or even included as a part of the therapeutical prescription. This concept should also be shared with patients, who often ask their doctor for diet and dietological suggestions to improve their skin quality. Nowadays, our data contributed to the literature to solve doubts regarding the potential modulation of skin biology that water induced; yet, at the same time, the data maintained that water is an active principle and factor to be considered in science and therapy.

In healthy patients, oligo-mineral water displayed a prominent effect in restoring the cutaneous barrier, as previously suggested in atopic dermatitis patients [13]. From this point of view, oligo-mineral water should always be supplemented in the case of barrier dysfunction or even allergic disorders.

At the same time, in line with Caiazzo et al.’s findings, our results could also suggest a direct effect on the skin microbiome capable of positively influencing the skin barrier and its lipidomics. Future efforts should be focused on a better understanding of the biological dynamics occurring between the cutaneous microbiome and keratinocytes biology. To confirm our data, omics and machine learning were able to magnify the water-based changes in cutaneous lipidomics and may project clinicians into the new future led by precision medicine.

Despite the innovative design (omics and machine learning) the present clinical trial also has some limitations, such as the inclusion of solely female subjects between 30 to 50 years of age. Thus, future studies should also validate these results in different ages, males and even diets.

## 5. Conclusions

Water orally consumed has a remarkable effect on the skin lipidomics in healthy subjects, so especially oligo-mineral water should be regarded as a complementary therapy in dermatological disorders. Furthermore, clinical trials evaluating nutritional supplements or diets in dermatology should account also for the water type consumed. Thus, water has to be regarded as a supplement for its biological effect on skin.

## Figures and Tables

**Table 1:** Instrumental details for cutaneous lipidomics analysis.

N.	Time	Flow (μL/min)	%A	%B
1	0.00	Run		
2	0.00	400	55.0	45.0
3	2.00	400	55.0	45.0
4	12.00	400	3.0	97.0
5	17.00	400	3.0	97.0
6	17.10	400	55.0	45.0
7	20.00	400	55.0	45.0
8	20.00	Stop run		

**Table 2:** Cutaneous lipids differentially changed after 1 month of Water A.

Formula	Metabolite Name	Ontology	Adduct Type	Fold Change	*p* Value
C_26_H_50_NO_7_P	LPC 18:2	LPC	[M + H]^+^	1.918	0.001
C_26_H_52_NO_7_P	LPC 18:1	LPC	[M + H]^+^	1.432	0.001
C_26_H_54_NO_7_P	LPC 18:0	LPC	[M + H]^+^	1.853	0.001
C_31_H_56_O_5_	DG 28:2|DG 14:1_14:1	DG	[M + NH_4_]^+^	2.180	0.001
C_31_H_58_O_5_	DG 28:1|DG 12:0_16:1	DG	[M + NH_4_]^+^	1.620	0.001
C_31_H_60_O_5_	DG 28:0|DG 12:0_16:0	DG	[M + NH_4_]^+^	1.652	0.001
C_33_H_64_O_4_	DG O-30:1|DG O-15:0_15:1	EtherDG	[M + NH_4_]^+^	14.417	0.001
C_34_H_64_O_4_	DG O-31:2|DG O-17:1_14:1	EtherDG	[M + NH_4_]^+^	5.561	0.001
C_34_H_66_O_4_	DG O-31:1|DG O-16:0_15:1	EtherDG	[M + NH_4_]^+^	11.102	0.001
C_32_H_60_O_6_	TG 29:0|TG 8:0_10:0_11:0	TG	[M + NH_4_]^+^	2.867	0.001
C_33_H_62_O_5_	DG 30:1	DG	[M + Na]^+^	1.212	0.001
C_34_H_54_O_5_	DG 31:6	DG	[M + Na]^+^	1.878	0.001
C_33_H_62_O_6_	TG 30:0|TG 10:0_10:0_10:0	TG	[M + Na]^+^	1.940	0.001
C_36_H_68_O_4_	DG O-33:2|DG O-17:1_16:1	EtherDG	[M + NH_4_]^+^	6.269	0.001
C_35_H_64_O_5_	DG 32:2	DG	[M + Na]^+^	9.000	0.001
C_37_H_68_O_4_	DG O-34:3|DG O-17:1_17:2	EtherDG	[M + NH_4_]^+^	7.566	0.001
C_37_H_70_O_4_	DG O-34:2|DG O-19:1_15:1	EtherDG	[M + NH_4_]^+^	7.996	0.001
C_36_H_66_O_5_	DG 33:2	DG	[M + Na]^+^	5.208	0.001
C_38_H_72_O_4_	DG O-35:2|DG O-19:1_16:1	EtherDG	[M + NH_4_]^+^	3.744	0.001
C_37_H_70_O_5_	DG 34:1	DG	[M + Na]^+^	1.064	0.001
C_38_H_68_O_5_	DG 35:3|DG 15:0_20:3	DG	[M + NH_4_]^+^	2.732	0.001
C_40_H_76_O_4_	DG O-37:2|DG O-19:1_18:1	EtherDG	[M + NH_4_]^+^	2.652	0.001
C_38_H_70_O_6_	TG 35:1|TG 8:0_10:0_17:1	TG	[M + NH_4_]^+^	3.620	0.001
C_40_H_78_O_4_	DG O-37:1|DG O-15:0_22:1	EtherDG	[M + NH_4_]^+^	3.468	0.001
C_39_H_70_O_5_	DG 36:3	DG	[M + Na]^+^	1.262	0.001
C_38_H_72_O_6_	TG 35:0|TG 8:0_10:0_17:0	TG	[M + NH_4_]^+^	3.330	0.001
C_40_H_76_O_6_	TG 37:0|TG 8:0_14:0_15:0	TG	[M + NH_4_]^+^	2.478	0.001
C_42_H_80_O_6_	TG 39:0|TG 12:0_13:0_14:0	TG	[M + NH_4_]^+^	1.839	0.001
C_45_H_80_O_4_	DG O-42:5|DG O-26:3_16:2	EtherDG	[M + NH_4_]^+^	5.756	0.001
C_43_H_78_O_6_	TG 40:2|TG 8:0_16:1_16:1	TG	[M + NH_4_]^+^	3.351	0.001
C_43_H_82_O_6_	TG 40:0|TG 12:0_12:0_16:0	TG	[M + NH_4_]^+^	−2.792	0.001
C_42_H_79_NO_9_	HexCer 36:2;3O|HexCer 18:2;2O/18:0;O	HexCer_HS	[M + H]^+^	2.489	0.001
C_46_H_86_O_6_	TG 43:1|TG 13:0_14:0_16:1	TG	[M + NH_4_]^+^	1.356	0.001
C_46_H_88_O_6_	TG 43:0|TG 13:0_14:0_16:0	TG	[M + NH_4_]^+^	2.181	0.001
C_42_H_78_NO_8_P	PC 34:3	PC	[M + H]^+^	3.238	0.001
C_49_H_90_O_6_	TG 46:2|TG 14:0_16:1_16:1	TG	[M + NH_4_]^+^	1.398	0.001
C_45_H_86_NO_8_P	PC 37:2|PC 15:1_22:1	PC	[M + H]^+^	2.673	0.001
C_53_H_84_O_5_	DG 50:10|DG 24:5_26:5	DG	[M + NH_4_]^+^	1.467	0.001
C_47_H_86_NO_8_P	PC 39:4	PC	[M + H]^+^	2.299	0.001
C_53_H_96_O_5_	DG 50:4|DG 16:1_34:3	DG	[M + NH_4_]^+^	1.197	0.001
C_53_H_100_O_5_	DG 50:2|DG 16:1_34:1	DG	[M + H]^+^	−1.180	0.001
C_52_H_100_O_6_	TG 49:0|TG 16:0_16:0_17:0	TG	[M + NH_4_]^+^	1.112	0.001
C_53_H_92_O_6_	TG 50:5|TG 16:1_16:1_18:3	TG	[M + NH_4_]^+^	−1.038	0.001
C_55_H_96_O_6_	TG 52:5|TG 16:1_18:1_18:3	TG	[M + NH_4_]^+^	1.021	0.001
C_56_H_100_O_6_	TG 53:4|TG 17:1_18:1_18:2	TG	[M + NH_4_]^+^	−1.114	0.001
C_18_H_37_NO_2_	SPB 18:1;2O—Sphingosine	Sph	[M + H]^+^	1.945	0.001
C_57_H_108_O_6_	TG 54:1|TG 18:0_18:0_18:1	TG	[M + NH_4_]^+^	−1.389	0.001
C_18_H_39_NO_2_	SPB 18:0;2O—Sphinganine	DHSph	[M + H]^+^	2.398	0.001
C_58_H_108_O_6_	TG 55:2|TG 23:0_16:1_16:1	TG	[M + NH_4_]^+^	1.699	0.001
C_58_H_112_O_6_	TG 55:0|TG 15:0_16:0_24:0	TG	[M + NH_4_]^+^	1.117	0.001
C_59_H_104_O_6_	TG 56:5|TG 16:1_18:1_22:3	TG	[M + NH_4_]^+^	1.001	0.001
C_59_H_108_O_6_	TG 56:3|TG 20:0_18:1_18:2	TG	[M + NH_4_]^+^	−1.311	0.001
C_59_H_112_O_6_	TG 56:1|TG 16:0_24:0_16:1	TG	[M + NH_4_]^+^	1.018	0.001
C_61_H_116_O_6_	TG 58:1|TG 16:0_24:0_18:1	TG	[M + NH_4_]^+^	1.032	0.001
C_63_H_96_O_6_	TG 60:13|TG 20:4_20:4_20:5	TG	[M + NH_4_]^+^	1.062	0.001
C_63_H_98_O_6_	TG 60:12|TG 20:4_20:4_20:4	TG	[M + NH_4_]^+^	−1.013	0.001
C_23_H_44_O_5_	DG 20:0	DG	[M + Na]^+^	2.008	0.001
C_25_H_50_NO_4_	CAR 18:0	CAR	[M]^+^	2.523	0.001
C_25_H_48_O_5_	DG 22:0|DG 10:0_12:0	DG	[M + NH_4_]^+^	−1.282	0.001
C_27_H_50_O_5_	DG 24:1|DG 8:0_16:1	DG	[M + NH_4_]^+^	4.595	0.001
C_24_H_50_NO_7_P	LPC 16:0	LPC	[M + H]^+^	1.607	0.001
C_29_H_54_O_5_	DG 26:1|DG 8:0_18:1	DG	[M + NH_4_]^+^	8.049	0.001
C_31_H_58_O_4_	DG O-28:2|DG O-11:0_17:2	EtherDG	[M + NH_4_]^+^	8.626	0.001
C_30_H_56_O_5_	DG 27:1|DG 13:0_14:1	DG	[M + NH_4_]^+^	3.118	0.001
C_31_H_60_O_4_	DG O-28:1|DG O-15:0_13:1	EtherDG	[M + NH_4_]^+^	4.586	0.001
C_26_H_48_NO_7_P	LPC 18:3	LPC	[M + H]^+^	1.072	0.001

CAR: Acylcarnitine, DG: Diacylglycerol, DHSph: Sphinganine, EtherDG: Ether-linked diacylglycerol, HexCer_HS: Hexosylceramide hydroxyfatty acid-sphingosine, LPC: Lysophophatidylcholine, PC: Phosphatidylcholine, Sph: Sphingosine, TG: Triacylglycerol.

**Table 3:** Cutaneous lipids capable to predict the water type consumed.

Formula	Metabolite Name	Ontology	Adduct Type
C_26_H_52_NO_7_P	LPC 18:1	LPC	[M + H]^+^
C_31_H_56_O_5_	DG 28:2|DG 14:1_14:1	DG	[M + NH_4_]^+^
C_31_H_58_O_5_	DG 28:1|DG 12:0_16:1	DG	[M + NH_4_]^+^
C_31_H_60_O_5_	DG 28:0|DG 12:0_16:0	DG	[M + NH_4_]^+^
C_33_H_64_O_4_	DG O-30:1|DG O-15:0_15:1	EtherDG	[M + NH_4_]^+^
C_34_H_54_O_5_	DG 31:6	DG	[M + Na]^+^
C_37_H_70_O_5_	DG 34:1	DG	[M + Na]^+^
C_40_H_76_O_4_	DG O-37:2|DG O-19:1_18:1	EtherDG	[M + NH_4_]^+^
C_40_H_78_O_4_	DG O-37:1|DG O-15:0_22:1	EtherDG	[M + NH_4_]^+^
C_39_H_70_O_5_	DG 36:3	DG	[M + Na]^+^
C_43_H_82_O_6_	TG 40:0|TG 12:0_12:0_16:0	TG	[M + NH_4_]^+^
C_45_H_86_NO_8_P	PC 37:2|PC 15:1_22:1	PC	[M + H]^+^
C_53_H_96_O_5_	DG 50:4|DG 16:1_34:3	DG	[M + NH_4_]^+^
C_52_H_100_O_6_	TG 49:0|TG 16:0_16:0_17:0	TG	[M + NH_4_]^+^
C_55_H_96_O_6_	TG 52:5|TG 16:1_18:1_18:3	TG	[M + NH_4_]^+^
C_18_H_39_NO_2_	SPB 18:0;2O—Sphinganine	DHSph	[M + H]^+^
C_58_H_108_O_6_	TG 55:2|TG 23:0_16:1_16:1	TG	[M + NH_4_]^+^
C_63_H_96_O_6_	TG 60:13|TG 20:4_20:4_20:5	TG	[M + NH_4_]^+^
C_63_H_98_O_6_	TG 60:12|TG 20:4_20:4_20:4	TG	[M + NH_4_]^+^
C_26_H_48_NO_7_P	LPC 18:3	LPC	[M + H]^+^

CAR: Acylcarnitine, DG: Diacylglycerol, DHSph: Sphinganine, EtherDG: Ether-linked diacylglycerol, LPC: Lysophophatidylcholine, TG: Triacylglycerol.

## Data Availability

The data are available in a publicly accessible repository.

## Supplementary Materials

The following supporting information can be downloaded at: https://www.mdpi.com/article/10.3390/biomedicines11041036/s1, Supplementary S1.
