# Supplementary material for: Water Is an Active Element: A Randomized Double-Blind Controlled Clinical Trial Comparing Cutaneous Lipidomics in Consumers Drinking Two Different Bicarbonate-Calcic Waters (Medium-Mineral vs. Oligo-Mineral)
Source: Biomedicines. 2023 Mar 27;11(4):1036. doi: 10.3390/biomedicines11041036 (PMC10135906; doi:10.3390/biomedicines11041036)
Supplement: Supplementary file 1 [file biomedicines-11-01036-s001.zip › biomedicines-2098431-supplementary.pdf]

Supplementary S1

"Water A[1]" still medium-mineral water: official analysis and information
Aspect and characteristics: clear, colorless, taste-free and odorless

<u>*Chemical analysis*</u>:
Total dry solids at 180° C (mg/l): 181,6
Electrical conductivity at 20° C (μs/cm): 297,6
pH: 7,61
Carbon dioxide free at source (mg/L): 7,33

<u>*Dissolved substances expressed in ions (mg/L)*</u>:
Calcium Ca^{++}: 60,36
Sodium Na^{+}: 3,87
Magnesium Mg^{++}: 3,73
Potassium K^{+}: 0,35
Stronzium Sr^{++}: 0,13
Bicarbonates HCO_3^{-}: 185,4
Sulphates SO_4^{--}: 7,54
Chlorides Cl^{-}: 7,34
Nitrates NO_3^{-}: 1,38
Fluorides F^{-}: 0,14
Silica SiO_2: 5,19

Microbiologically pure

[1] "Water A" is marketed under the name of "Rocchetta naturale"

Analysis performed by the Università degli Studi di Camerino, 7 June 2018. Authorised by Health Ministry decree D.D. N° 3294-191, dated 24 January 2000. Authorised by DDGR Umbria n° 7819, dated 22 September 2004. 1,500 QUALITY CONTROLS DAILY - QUALITY CONTROL SYSTEM UNI EN ISO 9001-15 CSQA n° 023

"Water B[2]" still oligo-mineral water: official analysis and information
Aspect and characteristics: clear, colorless, naturally effervescent and calcium bicarbonate

Chemical analysis:
Dry residue at 180° C (mg/L): 741
Electrical conductivity at 20° C (µs/cm): 1099
pH: 5,8

Dissolved substances expressed in ions (mg/L):
Calcium Ca^{++}: 175
Sodium Na^{+}: 64
Magnesium Mg^{++}: 26
Potassium K^{+}: 7,2
Bicarbonates $HCO3^{-}$: 578
Sulphates $SO4^{--}$: 92
Chlorides Cl^{-}: 74
Nitrates $NO3^{-}$: 6,5
Fluorides F^{--}: 1,0
Silica SiO_2: 8,8
Lithium: 0,16

Antimony: <0.0002 mg/L
Cyanide: <0.0010 mg/L

Microbiologically pure

[2] "Water B" is a general oligo-mineral water decided as comparator

Analysis performed by the Università degli Studi di Camerino, 28 June 2019. QUALITY CONTROL SYSTEM UNI EN ISO 9001:2015 CSQA n° 014
